# Enhancing Laying Hen Productivity and Health: Influence of Dietary Probiotic *Bacillus* Strains and Prebiotic *Saccharomyces cerevisiae* Yeast Cell Wall on Production Performance, Egg Quality, and Inflammatory Responses

**DOI:** 10.3390/ani15101398

**Published:** 2025-05-12

**Authors:** Zafar M. Hakami, Rashed A. Alhotan, Ali R. Al Sulaiman, Riyadh S. Aljumaah, Valentino Palombo, Mariasilvia D’Andrea, Abdulrahman S. Alharthi, Ala E. Abudabos

**Affiliations:** 1 Department of Animal Production, College of Food and Agriculture Sciences, King Saud University, P.O. Box 2460, Riyadh 11451, Saudi Arabia; 437105818@student.ksu.edu.sa (Z.M.H.); ralhotan@ksu.edu.sa (R.A.A.); rjumaah@ksu.edu.sa (R.S.A.); abalharthi@ksu.edu.sa (A.S.A.); 2 Environmental Protection Technologies Institute, Sustainability and Environment Sector, King Abdulaziz City for Science and Technology, P.O. Box 6086, Riyadh 11442, Saudi Arabia; arsuliman@kacst.gov.sa; 3 Department of Agricultural, Environmental and Food Sciences, Università degli Studi del Molise, Via De Sanctis snc, 86100 Campobasso, Italy; valentino.palombo@unimol.it (V.P.); dandrea@unimol.it (M.D.); 4 Department of Agriculture, School of Agriculture and Applied Sciences, Alcorn State University, 1000 ASU Drive, Lorman, MS 39096-7500, USA

**Keywords:** egg quality, inflammatory response, laying hens, performance, prebiotic, probiotic

## Abstract

To evaluate the influences of dietary *Bacillus*-based probiotics and yeast-based prebiotics over 16 weeks, 500 37-week-old Hisex white layers were indiscriminately assigned to five dietary groups: T1, control; T2, control + *Bacillus subtilis* (1.1 × 10^8^ CFU/kg); T3, control + *Bacillus subtilis* and *Bacillus licheniformis* (1.3 × 10^9^ CFU/kg); T4, control + *Bacillus coagulans* (1.0 × 10^9^ CFU/kg); and T5, control + *Saccharomyces cerevisiae* yeast cell wall (0.25 g/kg). Cumulatively (*p* < 0.001), T1 showed the highest feed consumption, while T4 and T5 had higher egg production than T1 and T3. T3 yielded the heaviest eggs, whereas T2 produced the lightest. Egg mass was higher in T3, T4, and T5, with all supplemented groups showing improved feed conversion ratios compared to the control. Furthermore, T5 showed a higher wet yolk weight (*p* < 0.001), while T2 and T5 had a higher dry albumin weight (*p* < 0.05). Additionally, T3, T4, and T5 reduced serum IL-1β (*p* < 0.05), IL-6 (*p* < 0.01), and TNF-α (*p* < 0.01), alongside elevated IL-10 levels (*p* < 0.01) relative to the control. Overall, the results suggest that dietary supplementation with multi-strain combinations and yeast-derived prebiotics can enhance both productivity and immune health in laying hens.

## Simple Summary

In response to growing concerns about antibiotic resistance, many countries have prohibited the use of antibiotics as growth promoters in animal production. While antibiotics in animal feed have traditionally accelerated growth and prevented disease, their widespread use has led to significant issues, such as the development of antibiotic-resistant bacteria and residues in animal products. This study explored the potential of dietary supplements, particularly various strains of *Bacillus* probiotics and a prebiotic derived from yeast cell walls, to serve as alternatives to antibiotics in laying hen diets. Conducted over 16 weeks with 500 hens, this study tested five different diet plans, analyzing their impact on hens’ production performance, egg quality, and immune responses. The results show that, cumulatively, supplementation with *Bacillus coagulans* and yeast cell wall improves egg production, while all supplements enhance feed efficiency compared to the standard diet. Additionally, these supplements were effective in augmenting anti-inflammatory cytokine production while diminishing pro-inflammatory cytokine activities in the circulation of hens, suggesting an overall improvement in the birds’ immune competence. The findings suggest that probiotics and prebiotics can effectively replace antibiotics in poultry diets, offering benefits for both industry practices and consumer health by reducing issues related to antibiotic resistance and residues.

## 1. Introduction

Antibiotic growth promoters (AGPs) have been widely used in the poultry industry to enhance growth rates, improve feed efficiency, and reduce disease prevalence [1]. However, the rising concerns over antibiotic resistance and the residual effects of antibiotics in animal products have prompted the exploration of alternative strategies to maintain and improve poultry health and productivity [2]. Dietary supplements, such as probiotics and prebiotics, have emerged as promising alternatives to AGPs. Among these, *Bacillus subtilis*, *Bacillus licheniformis*, and *Bacillus coagulans* have gained attention for their probiotic properties, while *Saccharomyces cerevisiae* yeast cell wall (YCW) is recognized for its prebiotic benefits [3].

*Bacillus subtilis* is a well-studied probiotic known for its robust nature and ability to form endospores, allowing it to survive harsh environmental conditions, such as the acidic environment of the gastrointestinal tract [4]. This bacterium produces various enzymes, such as proteases, amylases, and cellulases, which enhance nutrient digestion and absorption in the host [5]. Additionally, *Bacillus subtilis* has been shown to produce antimicrobial substances, including bacteriocins and lipopeptides, which inhibit the growth of pathogenic bacteria, promoting a healthier gut microbiota balance and reducing the incidence of gastrointestinal infections [6,7]. Dietary supplementation with *Bacillus subtilis* has been demonstrated to ameliorate hen performance and internal egg quality, while also enhancing nutrient retention throughout the entire production cycle [8]. Similarly, Guo et al. [9] reported that the long-term supplementation of *Bacillus subtilis* markedly enhanced the laying performance and egg quality of layers by positively modulating the cecal microbiota.

*Bacillus licheniformis*, another spore-forming bacterium, shares several beneficial properties with *Bacillus subtilis*. It is widely used in poultry diets due to its ability to produce enzymes such as proteases, amylases, and cellulases, which aid in the digestion of complex feed components [10]. This enzymatic activity not only improves nutrient availability but also reduces the viscosity of intestinal contents, promoting a stable and healthy intestinal environment [11]. *Bacillus licheniformis* has also been shown to generate antimicrobial peptides that inhibit the growth of pathogenic bacteria, contributing to a healthier gut microbiota balance, which is crucial for optimal immune function [12]. A previous study demonstrated that dietary inclusion of *Bacillus licheniformis* boosted egg production and quality of laying hens through enhancing intestinal barrier function, promoting systemic immunity, and regulating reproductive hormone secretions [13].

*Bacillus coagulans*, although less commonly studied compared to the other two species, holds considerable promise as a probiotic for poultry [14]. This bacterium is unique due to its ability to produce lactic acid and other organic acids, which can lower the pH of the gastrointestinal tract, creating an environment less favorable for pathogenic bacteria [15]. Additionally, *Bacillus coagulans* has been shown to enhance the production of short-chain fatty acids in the gut, which not only serve as an energy source for colonocytes but also contribute to the maintenance of a healthy and balanced gut microbiome while modulating the immune system [16]. The recent research conducted by Xu et al. [17] has shown that supplementing the diet of laying hens with *Bacillus coagulans* X26 during the peak laying phase enhanced production performance and egg quality through the modulation of intestinal flora composition and structure, along with augmented concentrations of short-chain fatty acids in the intestine. Interestingly, the combination of these *Bacillus* species in poultry diets offers a multifaceted approach to synergistically boost the performance and health of chickens. Recent findings suggest that a dietary blend of *Bacillus licheniformis* and *Bacillus subtilis* can serve as a viable alternative to flavomycin in enhancing the laying performance and egg quality of aging hens, likely due to improved cecal microbial diversity and intestinal morphological structure [18].

The cell wall of the yeast *Saccharomyces cerevisiae* is a complex structure composed primarily of mannoproteins, beta-glucans, and chitin. These components play a crucial role in the cell wall’s functionality and have been identified as key bioactive compounds with significant health benefits for animals [19]. Mannoproteins, which are glycoproteins present in the outer layer of the YCW, are known for their ability to agglutinate harmful bacteria, thereby preventing their attachment to the gut lining and reducing the risk of infection [20]. Beta-glucans, another major component of YCW, are polysaccharides that have garnered considerable interest due to their immunomodulatory properties. These molecules can enhance the innate immune system by stimulating the activity of macrophages, neutrophils, and natural killer cells [21]. Additionally, beta-glucans are recognized for their ability to bind to specific receptors on immune cells, thereby triggering a cascade of immune responses that enhance the host’s ability to fight off infections and inflammatory diseases [22]. Chitin, although present in smaller quantities compared to mannoproteins and beta-glucans, also contributes to the beneficial effects of YCW. Chitin and its derivatives, such as chitosan, have been shown to possess antimicrobial properties and the ability to modulate gut microbiota [23]. This modulation can lead to an increase in beneficial bacteria and a decrease in pathogenic microorganisms, promoting a healthier gut environment [24]. The combined effects of these bioactive components make YCW a potent prebiotic with the potential to enhance the productive performance and health of poultry. Previous research has shown that incorporating YCW into the diet of laying hens led to an augmented production performance, enhanced both internal and external egg quality, and played a role in better profitability [25]. Additionally, a recent study by Zhou et al. [26] found that dietary YCW polysaccharides not only enhanced productive performance but also mitigated immune and inflammatory stress responses in laying hens, whether challenged or not with *Escherichia coli* lipopolysaccharide, partially through the modulation of gut microbial composition.

Despite the promising results from individual studies, there is still a need for comprehensive research directly comparing these natural alternatives to AGPs, either individually or in combination, in terms of their effects on the performance and health conditions of laying hens. To address this gap, it has been hypothesized that supplementing laying hen diets with *Bacillus* spp.-based probiotics, or with YCW-based prebiotics, could improve productive performance and positively modulate immune response compared to a standard diet, leveraging various mechanisms. Therefore, the objective of this research was to assess and contrast the influence of these dietary supplements on the production efficiency, egg quality, and immune-related inflammatory cytokine responses in laying hens.

## 2. Materials and Methods

### 2.1. Experiment Design and Husbandry Practices

The animal study protocol was approved by the Ethics Committee of King Saud University, Riyadh, Saudi Arabia (KSU-SE-21-38).

In total, 500 Hisex white laying hens at 37 weeks old, with similar weights and laying rates, were accommodated in 25-floor cages, each measuring 1.9 m × 2.0 m. The hens were evenly and indiscriminately divided into five treatment groups, each comprising five replicates of 20 birds. The dietary interventions, summarized in Table 1, were as follows: T1 involved the control diet without any supplementation, T2 included the control diet supplemented with *Bacillus subtilis* (DSM17299) at a rate of 1.1 × 10^8^ CFU per kg, T3 comprised the control diet supplemented with *Bacillus subtilis* (DSM5750) and *Bacillus licheniformis* (DSM5749) in a 1:1 ratio, at a concentration of 1.3 × 10^9^ CFU per kg, T4 encompassed the control diet supplemented with *Bacillus coagulans* (DSM 32016) at a rate of 1.0 × 10^9^ CFU per kg, and T5 involved the control diet supplemented with *Saccharomyces cerevisiae* YCW at a rate of 0.25 g per kg. The supplement dosages, determined in accordance with the manufacturer’s guidelines, were added to the mixture, and the final inclusion levels were verified through analysis. The hens received their assigned diets for 16 weeks, ending the experiment when they reached 52 weeks of age.

Before commencing the experiment, a two-week pre-feeding trial was conducted to progressively acclimate the chickens to transition from a standard diet to the experimental diets. The control diet, relying on maize and soybean meal, was crafted following the NRC [27] to satisfy the nutritional demands of laying hens. The ingredients and nutritional content of the diet are outlined in Table 2. The nutrient content of the feed ingredients used in diet formulation was determined according to the methods outlined by AOAC [28]. The hens were reared in a climate-controlled environment with an average air temperature of 22 ± 2 °C, a relative humidity of 55 ± 5%, and subjected to a daily lighting cycle of 16 h at 15 lux intensity followed by 8 h of darkness. Throughout the trial, the birds were raised in accordance with the recommended managerial and hygienic standards and were provided ad libitum access to mash feed and clean water.

### 2.2. Sampling and Measurements

For the evaluation of production metrics over targeted timeframes (37–40, 41–44, 45–48, and 49–52 weeks) and cumulatively (37–52 weeks), daily recordings of egg production (EP) and egg weight (EW), alongside a weekly determination of feed intake (FI), were utilized to compute the egg mass (EM) and feed conversion ratio (FCR) for each replication. The EP, figured by dividing the count of eggs produced by the count of birds existing on the same day, in conjunction with EW, contributed to deriving the EM through the multiplication of the average EW by the rate of EP. Subsequently, the average FI, determined by deducting the leftover feed from the total portion provided, was divided by the EM to compute the FCR. Each replicate’s cage was monitored daily for mortality and abnormal egg production.

To assess egg quality traits, ten typical eggs were indiscriminately chosen from each treatment group at weeks 40, 44, 48, and 52 of the trial. After weighing the eggs, the shells were carefully removed, and the yolks and albumens were then separated utilizing an egg separator. The wet weightiness of the shells (WSs), yolks (WYs), and albumens (WAs) were recorded with a precision digital balance. Afterward, the separated portions were dried in a convection oven set to 105 °C for a 24 h period. After drying, they were cooled in a desiccator and weighed to determine the dry weights of the yolk (DY), albumen (DA), and shell (DS). The weights of egg portions, both wet and dry, were computed as proportions relative to the fresh egg weights.

Yolk color parameters were measured utilizing a chroma meter (CR-400, Konica Minolta, Tokyo, Japan), capturing values for L*, a*, and b*. The L* value indicates lightness, ranging from 0 (black) to 100 (white). The a* value reflects the red–green spectrum, with negative readings signifying green hues and positive readings indicating red tones. Similarly, the b* value corresponds to the yellow–blue axis, where negative readings correspond to blue and positive readings to yellow.

For the analysis of inflammatory cytokine secretion, ten hens were indiscriminately chosen from each treatment group at the conclusion of the experiment (52 weeks). Approximately 5 mL of blood was extracted from the brachial vein of each bird and promptly placed into a serum separator tube (BD Vacutainer; Becton, Dickinson and Company, Franklin Lakes, NJ, USA). The tubes underwent centrifugation at 3000 rpm for 10 min at 4 °C to acquire serum specimens, which were kept in sterilized 1.5 mL Eppendorf tubes at −80 °C pending analysis. The seral concentrations of interleukin-1 beta (IL-1β), interleukin-6 (IL-6), interleukin-10 (IL-10), interferon-gamma (IFN-γ), and tumor necrosis factor-alpha (TNF-α) were quantified utilizing ELISA kits (Thermo Fisher Scientific, Waltham, MA, USA), adhering to the manufacturer’s prescribed guidelines.

### 2.3. Data Analysis

This study employed a completely randomized design, with each replicate serving as the experimental unit. Before conducting the statistical analysis, both the homogeneity of variance and the normality of residuals were verified utilizing Levene’s test and Shapiro–Wilk’s test, respectively. The statistical analysis was performed by applying the generalized linear model of SAS (version 9.4; SAS Institute Inc., Cary, NC, USA), operating a one-way analysis of variance technique along with Tukey’s test for multiple mean comparisons. The Proc Mixed procedure of SAS was employed for analyzing repeated measurements of production performance and egg quality characteristics. A significance level of *p* < 0.05 was adopted. The findings are displayed as least-square means, accompanied by their corresponding pooled standard errors of the mean.

## 3. Results

### 3.1. Production Performance

**Figure 1:**
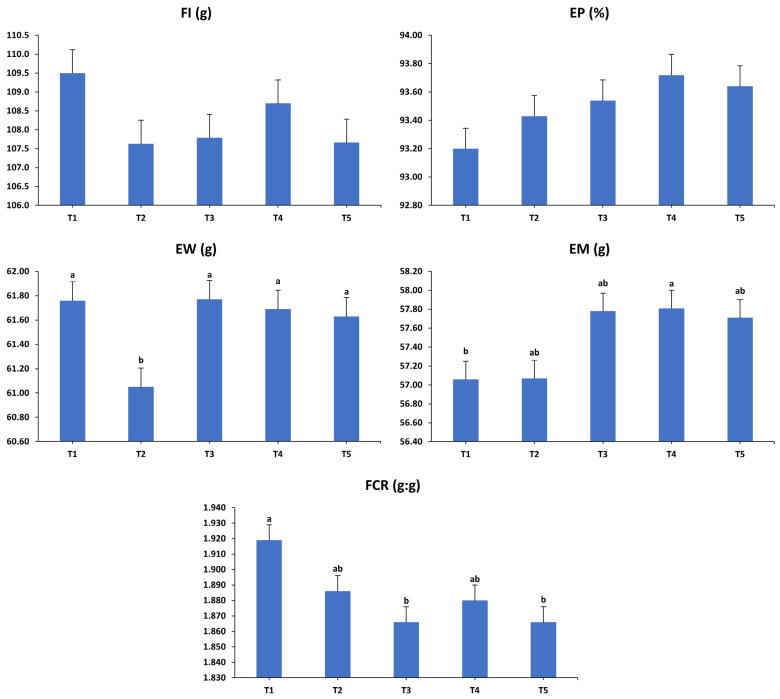
Influence of the dietary treatments on the production performance of laying hens at 37–40 weeks of age. FI: feed intake; EP: egg production; EW: egg weight; EM: egg mass; FCR: feed conversion ratio. T1: control diet; T2: control diet + *Bacillus subtilis*; T3: control diet + *Bacillus subtilis* and *Bacillus licheniformis*; T4: control diet + *Bacillus coagulans*; T5: control diet + yeast cell wall. Data were analyzed using one-way ANOVA and are presented as mean ± pooled standard error of the mean. Bars with different superscript letters, determined by Tukey’s test, indicate significant differences (*p* < 0.05).

The impacts of various dietary interventions on the laying hens’ productivity over the first (37–40 weeks) interval are shown in Figure 1. During the initial period, the analysis indicated that FI and EP remained unaffected by the dietary interventions (*p* > 0.05). However, significant changes were observed in EW, EM, and FCR (*p* < 0.01). Markedly, T2 recorded the lowest EW compared to the other groups. T4 demonstrated higher EM than T1, while T2, T3, and T5 showed intermediate values that did not differ significantly from T1 and T4. Furthermore, T1 exhibited a significantly less efficient FCR compared to T3 and T5, with T2 and T4 displaying intermediate efficiencies similar to the other groups.

**Figure 2:**
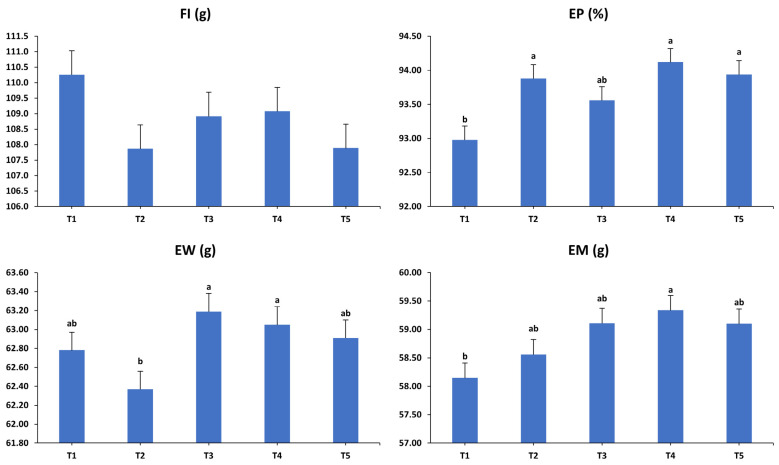

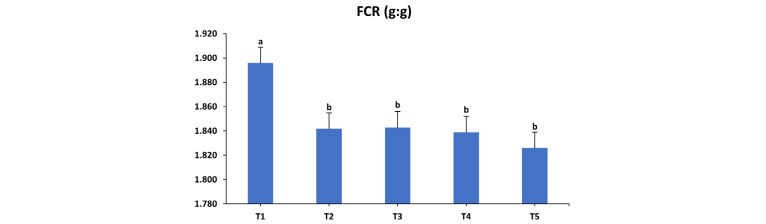
Influence of the dietary treatments on the production performance of laying hens at 41–44 weeks of age. FI: feed intake; EP: egg production; EW: egg weight; EM: egg mass; FCR: feed conversion ratio. T1: control diet; T2: control diet + *Bacillus subtilis*; T3: control diet + *Bacillus subtilis* and *Bacillus licheniformis*; T4: control diet + *Bacillus coagulans*; T5: control diet + yeast cell wall. Data were analyzed using one-way ANOVA and are presented as mean ± pooled standard error of the mean. Bars with different superscript letters, determined by Tukey’s test, indicate significant differences (*p* < 0.05).

The impacts of various dietary interventions on the laying hens’ productivity over the second (41–44 weeks) interval are shown in Figure 2. During the subsequent period, FI remained consistent and unaffected by dietary interventions (*p* > 0.05). In contrast, EP (*p* < 0.001), EW (*p* < 0.05), EM (*p* < 0.01), and FCR (*p* < 0.01) showed significant variations due to these interventions. EP was considerably higher in T2, T4, and T5 compared to T1, with T3 showing intermediate EP levels that did not significantly vary from the other groups. Hens in T3 and T4 displayed greater EWs compared to those in T2, while T1 and T5 had intermediate weights relative to the other treatment groups. As a result of these differences in the EP and EW, higher EM values were observed in hens from T4 compared to those in T1, while T2, T3, and T5 displayed intermediate EM values falling between those of T1 and T4. Importantly, the supplemented groups demonstrated a more efficient conversion of feed into EM compared to the unsupplemented control group.

**Figure 3:**
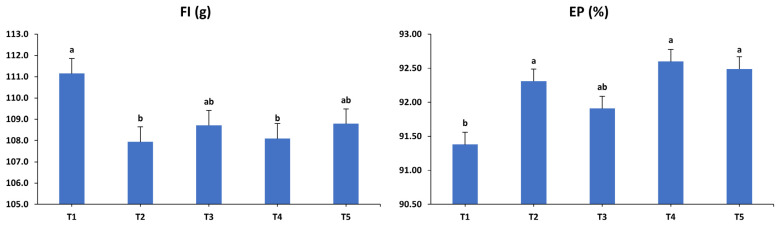

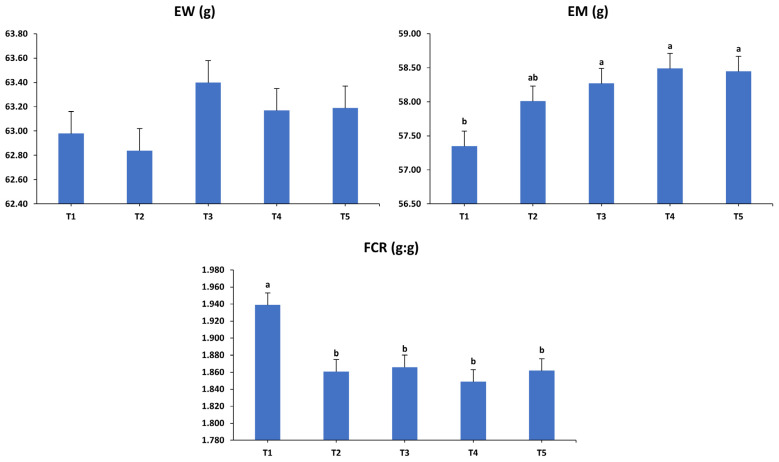
Influence of the dietary treatments on the production performance of laying hens at 45–48 weeks of age. FI: feed intake; EP: egg production; EW: egg weight; EM: egg mass; FCR: feed conversion ratio. T1: control diet; T2: control diet + *Bacillus subtilis*; T3: control diet + *Bacillus subtilis* and *Bacillus licheniformis*; T4: control diet + *Bacillus coagulans*; T5: control diet + yeast cell wall. Data were analyzed using one-way ANOVA and are presented as mean ± pooled standard error of the mean. Bars with different superscript letters, determined by Tukey’s test, indicate significant differences (*p* < 0.05).

The impacts of dietary interventions on the productive performance of laying hens during the third (45–48 weeks) interval are exhibited in Figure 3. Significant influences of the dietary treatments were observed on FI (*p* < 0.05), EP (*p* < 0.001), EM (*p* < 0.01), and FCR (*p* < 0.001) during the third period. Specifically, FI was lower in T2 and T4 compared to T1, while T3 and T5 showed intermediate FI levels that did not vary considerably from those observed in the other groups. T2, T4, and T5 exhibited increased EP compared to T1, whereas T3 displayed an EP rate comparable to the other treatment groups. Additionally, EM values were higher in hens from T3, T4, and T5 compared to those in T1, while T2 showed no significant difference in EM relative to the other groups. Similar to the previous period, all supplemented diets resulted in better FCRs compared to the unsupplemented control diet. However, EW was unaffected by any of the treatments during this period (*p* > 0.05).

**Figure 4:**
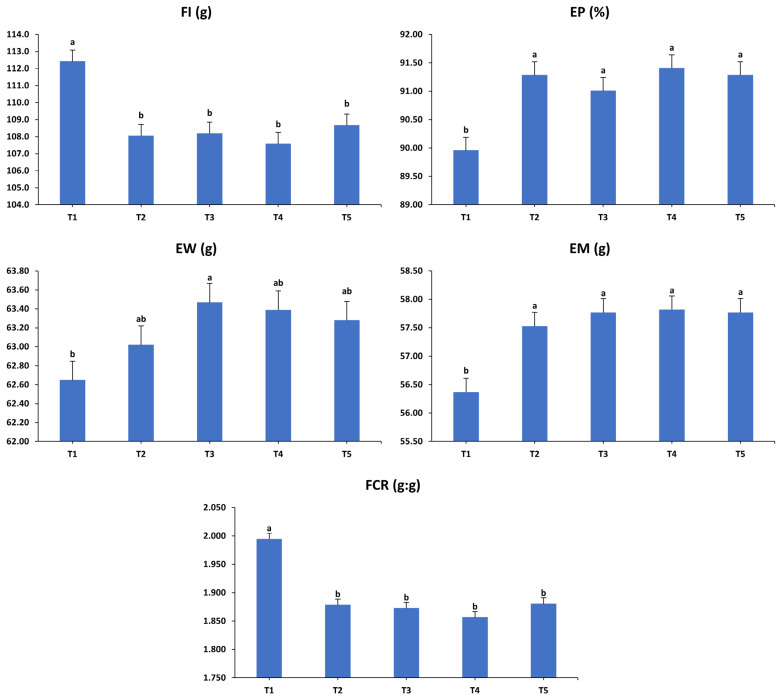
Influence of the dietary treatments on the production performance of laying hens at 49–52 weeks of age. FI: feed intake; EP: egg production; EW: egg weight; EM: egg mass; FCR: feed conversion ratio. T1: control diet; T2: control diet + *Bacillus subtilis*; T3: control diet + *Bacillus subtilis* and *Bacillus licheniformis*; T4: control diet + *Bacillus coagulans*; T5: control diet + yeast cell wall. Data were analyzed using one-way ANOVA and are presented as mean ± pooled standard error of the mean. Bars with different superscript letters, determined by Tukey’s test, indicate significant differences (*p* < 0.05).

The impacts of dietary interventions on the productive performance of laying hens during the fourth (49–52 weeks) interval are exhibited in Figure 4. During the fourth period, the dietary treatments significantly influenced all assessed performance metrics of the laying hens. Hens in T1 consumed more feed than those in all other treatments (*p* < 0.001) and correspondingly produced fewer eggs compared to hens on supplemented diets (*p* < 0.001). EW was considerably higher in hens that received T3 compared to those given T1, while those receiving T2, T4, and T5 demonstrated intermediate weights between T1 and T3 (*p* < 0.05). Moreover, EM was significantly higher in the supplemented groups than in the unsupplemented control group (*p* < 0.001). Likewise, hens fed the supplemented diets exhibited greater efficiency in converting feed into EM compared to those on the unsupplemented control diet (*p* < 0.001).

**Figure 5:**
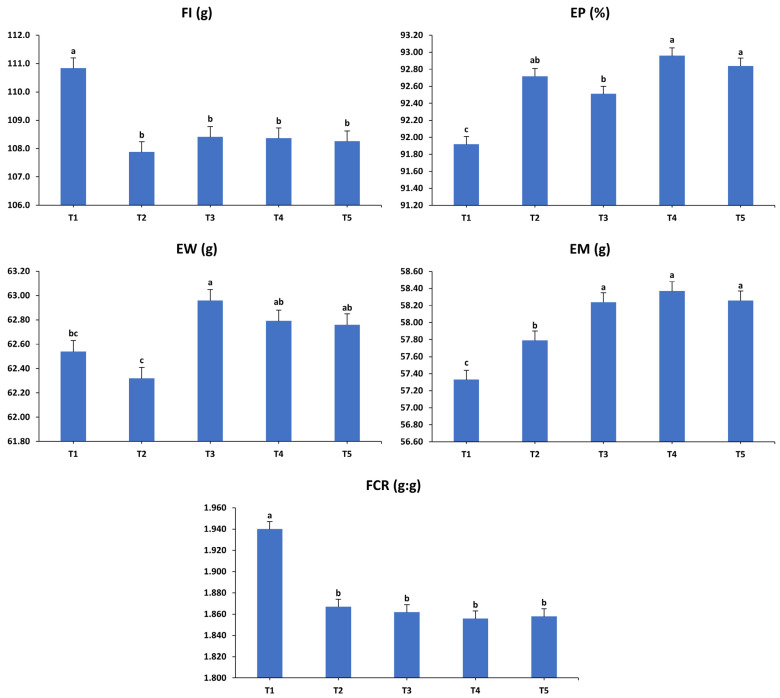
Influence of dietary treatments on the cumulative performance of laying hens aged 37–52 weeks. FI: feed intake; EP: egg production; EW: egg weight; EM: egg mass; FCR: feed conversion ratio. T1: control diet; T2: control diet + *Bacillus subtilis*; T3: control diet + *Bacillus subtilis* and *Bacillus licheniformis*; T4: control diet + *Bacillus coagulans*; T5: control diet + yeast cell wall. Data were analyzed using one-way ANOVA and are presented as mean ± pooled standard error of the mean. Bars with different superscript letters, determined by Tukey’s test, indicate significant differences (*p* < 0.05).

The cumulative influences of dietary regimens on various performance metrics of laying hens over a 16-week duration (37–52 weeks) are shown in Figure 5. The analysis indicated no significant two-way interactions between dietary regimens and temporal intervals for any performance indicators (*p* > 0.05). Cumulatively, hens receiving the T1 diet consumed more feed than those in all other treatments, showing an average increase in consumption of 2 g per day (*p* < 0.001). Hens fed T4 and T5 diets exhibited greater EP rates than those on T1 or T3 diets, with the T1 group exhibiting the lowest EP rate among all groups, while T2 had an intermediary rate that was not significantly different from T3, T4, and T5 (*p* < 0.001). Regarding EW, the heaviest eggs were produced by T3 and the lightest by T2, with T4 and T5 falling between T1 and T3, while T1 was positioned between T2, T4, and T5 (*p* < 0.001). The EM, influenced by EP and EW, exhibited significantly higher values in eggs from T3, T4, and T5 compared to those from T1 and T2, with T1 showing the lowest mass (*p* < 0.001). The efficiency of converting feed into EM significantly improved across all supplemented groups compared to the unsupplemented control group (*p* < 0.001).

**Figure 6:**
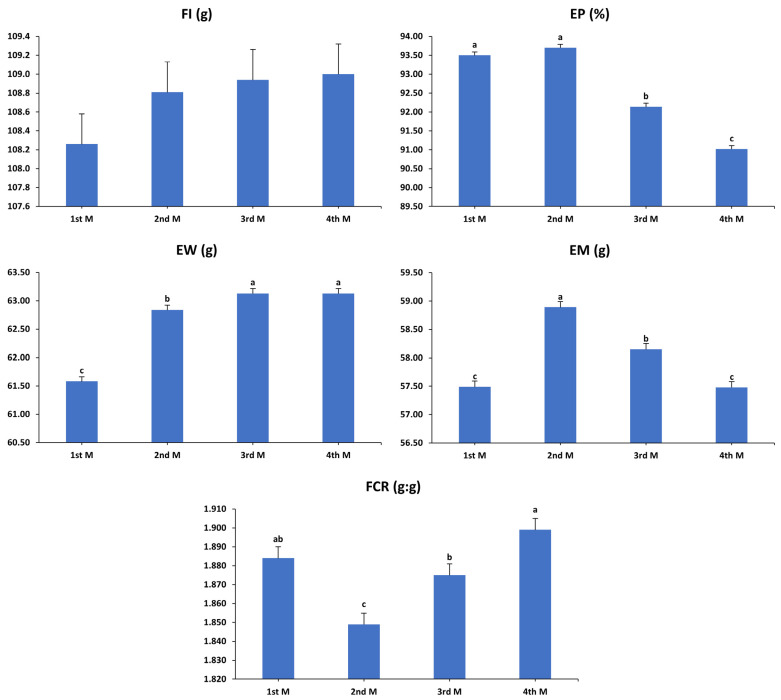
Influence of temporal intervals (in months) on the cumulative performance of laying hens aged 37–52 weeks. The data were analyzed to evaluate the impact of various temporal intervals or hen age (spanning the first through fourth months) within the laying cycle on cumulative performance, independent of the effects of dietary treatments. FI: feed intake; EP: egg production; EW: egg weight; EM: egg mass; FCR: feed conversion ratio. T1: control diet; T2: control diet + *Bacillus subtilis*; T3: control diet + *Bacillus subtilis* and *Bacillus licheniformis*; T4: control diet + *Bacillus coagulans*; T5: control diet + yeast cell wall. 1st M: first month; 2nd M: second month; 3rd M: third month; 4th M: fourth month. Data were analyzed using one-way ANOVA and are presented as mean ± pooled standard error of the mean. Bars with different superscript letters, determined by Tukey’s test, indicate significant differences (*p* < 0.05).

The cumulative influences of temporal intervals on various performance metrics of laying hens over a 16-week duration (37–52 weeks) are shown in Figure 6. When analyzing the data to evaluate how different temporal intervals within the hens’ laying cycle affected the cumulative performance, regardless of dietary treatments, no significant variation in the FI was observed across these intervals (*p* > 0.05). However, EP was considerably higher during the first two months compared to the last two months, with production in the third month exceeding that of the fourth (*p* < 0.001). In contrast, the later months (third and fourth) exhibited higher EW values compared to the earlier months (first and second), with the first month recording the lowest EW relative to the others (*p* < 0.001). EM showed a significant reduction in the first and fourth months compared to the second and third months, with the peak observed in the second month (*p* < 0.001). The most efficient FCR was observed in the second month, while the lowest efficiency occurred in the fourth month, with an intermediate ratio recorded in the first month, similar to that observed in the third and fourth months (*p* < 0.001).

### 3.2. Egg Quality Characteristics

**Figure 7:**
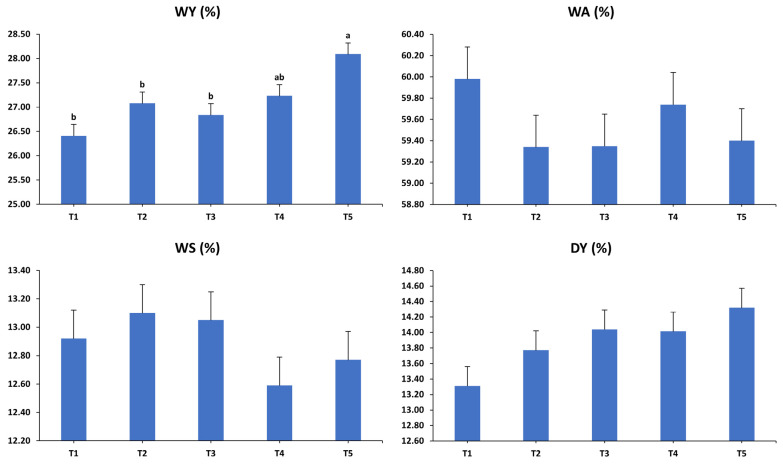

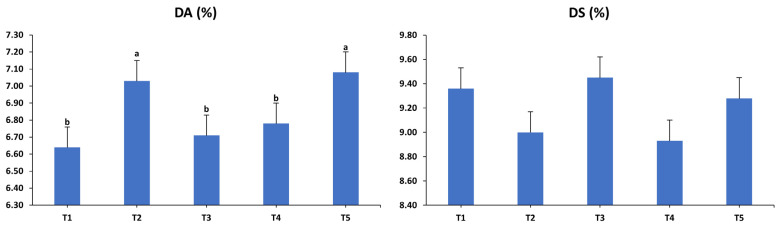
Influence of dietary treatments on egg breakout analysis (expressed as a percentage of egg weight) of laying hens at 40, 44, 48, and 52 weeks of age. WY: wet yolk; WA: wet albumin; WS: wet shell; DY: dry yolk; DA: dry albumin; DS: dry shell. T1: control diet; T2: control diet + *Bacillus subtilis*; T3: control diet + *Bacillus subtilis* and *Bacillus licheniformis*; T4: control diet + *Bacillus coagulans*; T5: control diet + yeast cell wall. Data were analyzed using one-way ANOVA and are presented as mean ± pooled standard error of the mean. Bars with different superscript letters, determined by Tukey’s test, indicate significant differences (*p* < 0.05).

The impacts of dietary interventions on egg breakout analysis in laying hens aged 40, 44, 48, and 52 weeks are displayed in Figure 7. The analysis revealed no significant two-way interactions between dietary interventions and sampling periods for any egg quality measurements (*p* > 0.05). Conversely, dietary interventions markedly affected the relative weights of WY (*p* < 0.001) and DA (*p* < 0.05). Eggs laid by hens on the T5 diet showed a higher percentage of WY than those on the T1, T2, and T3 diets, while eggs from the T4 group showed a similar percentage to the other intervention groups. Additionally, eggs from hens fed the T5 and T2 diets exhibited a higher DA percentage compared to those from hens on the T1, T3, and T4 diets. Numerically, eggs from the T5 group tended to have a higher DY percentage than all other interventions (*p* = 0.054). However, the relative weights of WA, WS, and DS were not significantly affected by the dietary interventions (*p* > 0.05).

**Figure 8:**
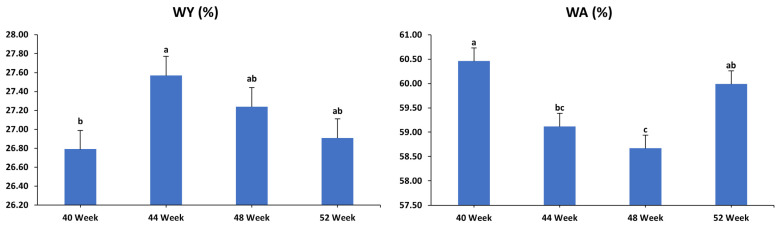

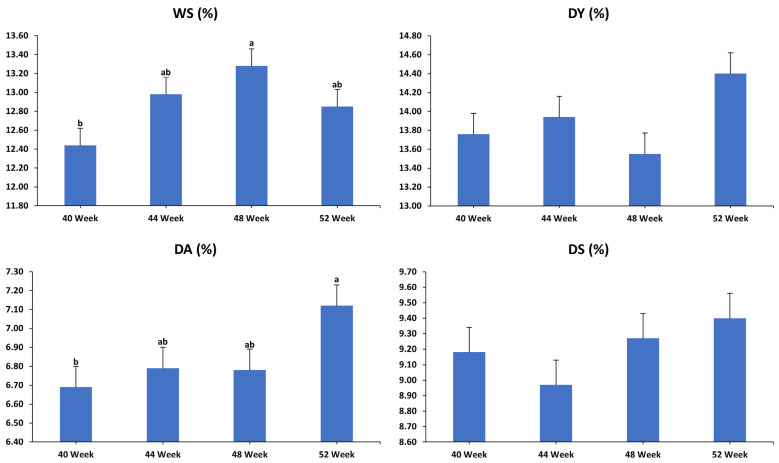
Influence of sampling periods (in weeks) on egg breakout analysis (expressed as a percentage of egg weight) of laying hens at 40, 44, 48, and 52 weeks of age. The data were aggregated to assess the impact of sampling periods on egg quality analysis, independent of the effects of dietary treatments. WY: wet yolk; WA: wet albumin; WS: wet shell; DY: dry yolk; DA: dry albumin; DS: dry shell. T1: control diet; T2: control diet + *Bacillus subtilis*; T3: control diet + *Bacillus subtilis* and *Bacillus licheniformis*; T4: control diet + *Bacillus coagulans*; T5: control diet + yeast cell wall. Data were analyzed using one-way ANOVA and are presented as mean ± pooled standard error of the mean. Bars with different superscript letters, determined by Tukey’s test, indicate significant differences (*p* < 0.05).

The impacts of sampling periods on egg breakout analysis in laying hens aged 40, 44, 48, and 52 weeks are displayed in Figure 8. When the data were aggregated to investigate the impact of sampling periods on egg quality analysis, irrespective of dietary treatments, it was found that the percentages of DY and DS showed no significant variation across sampling spans (*p* > 0.05). Conversely, the percentages of WY, WA, WS, and DA showed a significant variation depending on the time of sampling (*p* < 0.05; *p* < 0.001; *p* < 0.05; *p* < 0.05, respectively). Specifically, the percentage for WY peaked during the 44-week period compared to the 40-week period, while the values for the 48- and 52-week periods were intermediate and did not significantly differ from those of the earlier periods. Eggs from 40-week-old hens had the highest WA percentage, while those from 48-week-old hens had the lowest, with the percentage for 44 weeks falling between those of 48 and 52 weeks, and the 52-week eggs positioned between those of 40 and 44 weeks. In contrast, the percentage of WS was higher in 48-week-old hens compared to those aged 40 weeks, with intermediate percentages observed at 44 and 52 weeks that did not differ from those recorded during the other two periods. Furthermore, the proportion of DA was significantly lower for hens at 40 weeks compared to those at 52 weeks, while the percentages at 44 and 48 weeks were intermediate and showed no significant variation from the values observed during other sampling intervals.

The analysis of the egg-yolk-color index indicated that there were no significant differences between the dietary treatments, sampling intervals, or their interaction at 40, 44, 48, and 52 weeks of age (*p* > 0.05).

### 3.3. Immune-Related Inflammatory Response

**Figure 9:**
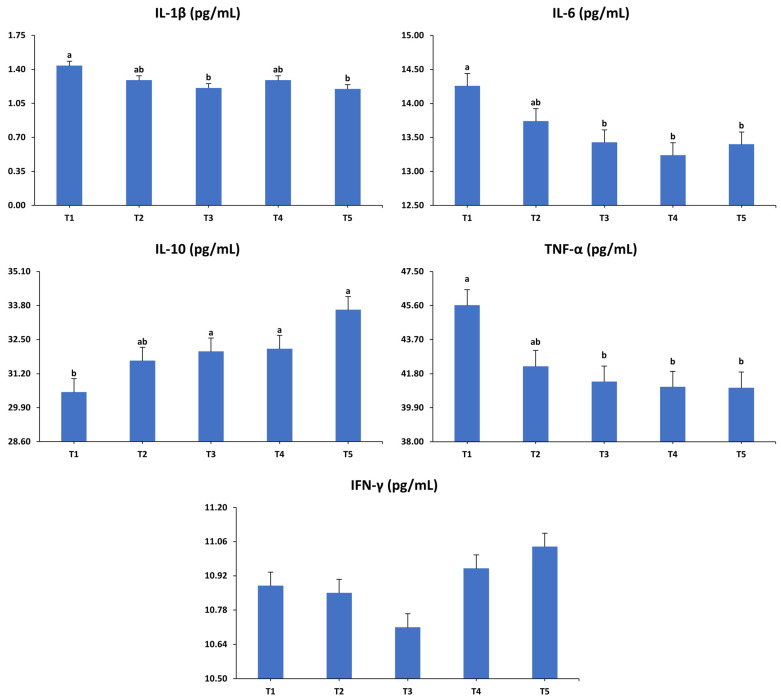
Influence of the dietary treatments on serum inflammatory cytokine levels in laying hens at 52 weeks of age. IL-1β: interleukin-1 beta; IL-6: interleukin-6; IL-10: interleukin-10; IFN-γ: interferon-gamma; TNF-α: tumor necrosis factor-alpha. T1: control diet; T2: control diet + *Bacillus subtilis*; T3: control diet + *Bacillus subtilis* and *Bacillus licheniformis*; T4: control diet + *Bacillus coagulans*; T5: control diet + yeast cell wall. Data were analyzed using one-way ANOVA and are presented as mean ± pooled standard error of the mean. Bars with different superscript letters, determined by Tukey’s test, indicate significant differences (*p* < 0.05).

The impacts of the dietary interventions on the levels of anti-inflammatory and pro-inflammatory cytokines in laying hens at 52 weeks of age are shown in Figure 9. The findings reveal significant impacts of the dietary treatments on serum concentrations of IL-1β (*p* < 0.05), IL-6 (*p* < 0.01), TNF-α (*p* < 0.01), and IL-10 (*p* < 0.01). Specifically, IL-1β levels were lower in hens fed with the T3 and T5 diets compared to those on the T1 diet, while the T2 and T4 groups showed intermediate levels that did not significantly differ from the other groups. Likewise, hens in T3, T4, and T5 exhibited lower IL-6 concentrations compared to those from T1, whereas the T2 group showed an intermediary level with no significant differences relative to the other treatment groups. Furthermore, hens fed the T3, T4, and T5 diets demonstrated reduced levels of TNF-α relative to those on the T1 diet, while the TNF-α level in the T2 group was intermediate, falling between the other groups. On the contrary, the concentration of IL-10 significantly augmented in T3, T4, and T5 compared to T1, with T2 exhibiting an intermediate level that did not differ significantly from the other treatments. However, no significant effect of the dietary treatments on IFN-γ concentration was observed (*p* > 0.05).

## 4. Discussion

This study investigated the cumulative effects of various dietary treatments, comprising microbial and yeast-based feed additives, along with temporal intervals, on the productive performance of laying hens from 37 to 52 weeks of age, offering insights into the functional benefits of these additives in improving poultry productivity. The absence of significant interactions between dietary treatments and temporal intervals suggests that while both factors independently influenced performance metrics, their combined effects did not exhibit synergistic or antagonistic outcomes. The significantly higher FI in the control group (T1) compared to all supplemented groups indicates a potential inefficiency in nutrient utilization when no additive is included. In contrast, the reduced FI in T2–T5 indicates improved nutrient assimilation, likely resulting from the modulatory effects of probiotics and yeast cell wall components on the intestinal microbiota. These findings resonate with the conclusions of Khomayezi and Adewole [29], who reported that pro/prebiotic supplementation enhances nutrient digestibility and gut health, potentially reducing the FI while maintaining or improving the performance outcomes. Notably, hens fed T4 (*Bacillus coagulans*) and T5 (yeast cell wall) exhibited superior EP values, while those on the T1 diet had the poorest output. These findings suggest a positive impact of both feed additives on laying persistence and reproductive efficiency, which is consistent with Xu et al. [30], who demonstrated that supplementary probiotics could modulate gut microbiota, immune function, and hormonal responses to enhance laying performance. Similarly, yeast cell wall components, rich in beta-glucans and mannan-oligosaccharides, are known for their immunomodulatory and gut barrier-enhancing effects, contributing to sustained production rates, as reported by Youssef et al. [31] and Alqhtani et al. [32].

Interestingly, T3 (a combination of *Bacillus subtilis* and *Bacillus licheniformis*) produced the heaviest eggs, while T2 (*Bacillus subtilis* alone) resulted in the lightest, despite similar EP rates. This suggests that strain specificity and synergistic effects between bacterial species may play a critical role in modulating nutrient partitioning towards egg mass rather than egg number. This phenomenon has been explored in part by Upadhaya et al. [33], who found that multi-strain probiotics can exert more profound effects on metabolite profiles and nutrient absorption than single-strain formulations. Egg mass (EM), as an integrated measure of both EP and EW, was maximized in T3, T4, and T5, indicating that these dietary treatments offered the most balanced support for both quantity and quality of egg production. T1 and T2, conversely, were less effective, underscoring their limited ability to meet the physiological demands of hens and to sustain consistent overall productivity during the laying period. Furthermore, FCR was significantly improved in all supplemented groups compared to T1, indicating that dietary supplementation enhanced the hens’ ability to convert feed into EM efficiently. This improvement reflects not only better nutrient absorption but possibly also reduced maintenance energy requirements due to improved gut health and metabolic efficiency [34]. This agrees with the findings of Pan et al. [35] and Wang et al. [36], who reported that dietary supplementation with probiotic *Bacillus* spp. strains could enhance intestinal health, boost digestive enzyme activity, and modulate immune responses in hens, ultimately leading to improved nutrient conversion into egg mass (EM).

The temporal analysis revealed that laying performance declined as hens aged from 37 to 52 weeks. While FI remained constant over time, EP was significantly higher during the first two months (37–40 weeks) than in the latter two (49–52 weeks), with a marked drop observed in the fourth month. This decline is consistent with established laying cycles, where hens exhibit peak production around 35–45 weeks, followed by a gradual decrease due to age-related reproductive senescence [37]. In contrast, EW increased over time, with the highest weights observed in the third and fourth months. This inverse relationship between EP and EW is a well-documented phenomenon, often attributed to physiological shifts wherein fewer eggs are produced but more yolk material is deposited per egg as hens age [38]. Interestingly, EW showed the opposite trend, with higher values observed in the later months (third and fourth). This could reflect an increase in egg size as hens continue laying, possibly due to changes in the ovarian or oviductal conditions as hens age [39]. The relationship between EP and EW is complex, as an increase in EW can sometimes be accompanied by a decrease in overall production, as observed in our study. However, this trade-off between egg number and size is a well-documented phenomenon in commercial layers [40]. The optimal FCR observed in the second month coincided with the peak in egg mass (EM), indicating that this period likely represents the physiological and productive apex for hens within this age range. In contrast, the poorest FCR recorded in the fourth month suggests a decline in production efficiency, potentially attributable to age-related physiological changes and reduced nutrient utilization, where feed intake is no longer effectively translated into output.

Notably, the inclusion of yeast cell wall (T5) significantly elevated the percentage of WY and DA, indicating a potential improvement in both yolk hydration and protein retention. These findings align with previous reports that yeast-derived products enhance gut health and nutrient absorption [41], potentially translating into better yolk formation and protein synthesis in the albumin [42]. The elevated DA content in both T5 and T2 groups (the latter supplemented with *Bacillus subtilis*) may reflect improved protein metabolism and structural integrity of the albumin, possibly driven by enzymatic activities or immunomodulatory effects associated with these feed additives [43]. The numerical increase in DY in the T5 group, although not statistically significant, suggests a trend towards better yolk dry matter deposition. Previous literature has emphasized the role of yeast-derived mannans and β-glucans in improving nutrient assimilation and modulating intestinal microbiota [44], which may underline the observed improvements in yolk quality.

Temporal changes in egg composition further highlight the physiological dynamics associated with hen aging. The peak in the WY percentage at 44 weeks, followed by intermediate values at 48 and 52 weeks, may be attributed to transient improvements in lipid synthesis or vitellogenesis as hens reach mid-lay. This echoes earlier findings by Marzec et al. [45], who noted fluctuations in yolk-to-albumin ratios as hens age, reflecting changes in hepatic lipoprotein production. Wet albumin (WA) percentage declined progressively with age, the lowest at 48 weeks, which may indicate a gradual decline in albumin synthesis or alterations in the water-binding capacity of the egg white. This mirrors patterns observed in older hens, which showed reduced albumin height and viscosity, impacting functional properties, like foaming and emulsification [45]. In contrast, the increase in WS at 48 weeks could indicate a transient improvement in shell formation, potentially linked to calcium metabolism efficiency or shell gland performance. However, since DS did not significantly vary, this WS increase might be due to temporary increases in moisture retention within the shell structure, rather than enhanced mineralization. Similarly, the increasing trend of DA with age, particularly the significant difference between 40 and 52 weeks, may reflect cumulative changes in albumen composition or alterations in the water/protein ratio due to age, as older hens often exhibit decreased water content in the albumen, resulting in a relatively higher concentration of dry albumen solids [46].

The significant reduction in pro-inflammatory cytokines IL-1β, IL-6, and TNF-α in the T3 (*Bacillus subtilis* + *Bacillus licheniformis*) and T5 (yeast cell wall) groups, as well as partially in the T4 (*Bacillus coagulans*) group, suggests a potential immunomodulatory capacity of these dietary interventions. IL-1β and TNF-α are key mediators in the acute phase of inflammation, while IL-6 is involved in both pro-inflammatory and anti-inflammatory pathways [47]. Their reduction in these groups suggests a systemic anti-inflammatory shift, which aligns with the previous findings demonstrating that *Bacillus*-based probiotics can attenuate inflammatory responses by enhancing gut barrier integrity and modulating immune signaling pathways [48]. Moreover, the observed elevation in IL-10, an anti-inflammatory cytokine, in T3, T4, and T5 supports the hypothesis that these treatments do not merely suppress inflammation but actively promote immune regulation. Notably, the combination of *Bacillus subtilis* and *Bacillus licheniformis* in the T3 diet elicited the most consistent decreases across IL-1β, IL-6, and TNF-α, along with a marked increase in IL-10, suggesting a possible synergistic interaction. This finding corroborates with Xu et al. [49], who reported enhanced anti-inflammatory effects when these two *Bacillus* strains were co-administered, potentially due to complementary enzyme production and improved colonization in the gut microbiota. However, further detailed immunological studies, including challenge trials assessing cytokine responses and leukocyte dynamics in relevant tissues, are warranted to confirm their immunological efficacy.

Similarly, the yeast cell wall in T5 appeared to exert a significant anti-inflammatory influence. Bioactive components, such as β-glucans and mannan-oligosaccharides in yeast cell walls, are recognized by pattern recognition receptors, like Dectin-1 and toll-like receptors, which can shift the immune response toward regulatory pathways [50]. The concurrent reduction in IL-1β, IL-6, and TNF-α, accompanied by the heightened concentration of IL-10 observed in T5 hens, supports this immunomodulatory role and aligns with previous findings in laying hens [26]. The intermediate cytokine levels observed in the T2 group (*Bacillus subtilis* only) suggest that while *Bacillus subtilis* alone may confer some benefit, it may be less effective than when used in combination with other strains. This observation is consistent with the growing body of literature supporting multi-strain probiotics as more efficacious due to their broader spectrum of action on host physiology and microbiota diversity [51]. Contrary to the other cytokines, no significant differences were found in IFN-γ levels across treatments. IFN-γ, a key Th1 cytokine, is often associated with responses to intracellular pathogens and cellular immunity [52]. The lack of variation suggests that the dietary treatments primarily modulated innate and regulatory inflammatory pathways rather than Th1-mediated responses. This aligns with the findings of Yosi and Metzler-Zebeli [53], who indicated that dietary probiotics had minimal effects on IFN-γ in chickens, unless the birds were exposed to immune challenges.

## 5. Conclusions

Dietary supplementation significantly influenced laying hen performance from 37 to 52 weeks of age. The inclusion of *Bacillus coagulans* (T4) and yeast cell wall (T5) notably enhanced EP, whereas the combination of *Bacillus subtilis* and *Bacillus licheniformis* (T3) increased the EW and overall EM. All supplemented groups (T2–T5) exhibited reduced FIs and improved FCRs, reflecting enhanced nutrient utilization efficiency compared to the control (T1). Dietary yeast cell wall supplementation (T5) improved the WY and DA percentages, suggesting better yolk hydration and protein retention. Additionally, dietary interventions with multi-strain probiotics (T3) and yeast-derived products (T5) significantly reduced pro-inflammatory cytokines (IL-1β, IL-6, TNF-α) and increased anti-inflammatory IL-10 levels, highlighting their superior immunomodulatory properties compared to single-strain probiotics (T2 and T4), likely due to synergistic interactions among microbial strains or bioactive components of yeast cell walls. Temporal trends revealed a decline in egg production over time, accompanied by increased egg weight, highlighting physiological trade-offs during aging. Overall, the results suggest that dietary supplementation with probiotics and yeast cell wall components can enhance both productivity and immune health in laying hens, with the best outcomes observed in multi-strain combinations and yeast-derived prebiotics. Future studies could build on these findings by incorporating additional biomarkers, assessing gene expression in gut tissues, analyzing microbiome composition, and evaluating immune responses under pathogen challenge conditions to elucidate the mechanisms underlying these dietary effects.

## Figures and Tables

**Table 1:** Summary of dietary interventions and supplementation levels.

Treatment	Diet Description
T1	Control diet without supplementation
T2	Control diet + *Bacillus subtilis* (DSM17299) at 1.1 × 10^8^ CFU/kg
T3	Control diet + *Bacillus subtilis* (DSM5750) and *Bacillus licheniformis* (DSM5749) in a 1:1 ratio at 1.3 × 10^9^ CFU/kg
T4	Control diet + *Bacillus coagulans* (DSM 32016) at 1.0 × 10^9^ CFU/kg
T5	Control diet + *Saccharomyces cerevisiae* yeast cell wall at 0.25 g/kg

**Table 2:** Composition and nutrient levels of the control diet (as-fed basis).

Items	Proportion, g/kg	Nutrients	Levels
Maize	556	Metabolic energy, MJ/kg	11.5
Soybean meal	276	Crude protein, g/kg	170
Wheat bran	25.0	Available phosphorus, g/kg	4.6
Maize oil	20.0	Calcium, g/kg	40
Di-calcium phosphate	16.0	Lysine, g/kg	7.6
Limestone	100	Methionine + Cysteine, g/kg	6.8
Sodium chloride	3.00	Threonine, g/kg	5.8
DL-methionine	2.00		
Vitamin–mineral mix ^1^	2.00		

^1^ Supplement provided the following per kilogram of diet: vitamin A (trans-retinyl acetate), 6600 IU; vitamin D3 (cholecalciferol), 2695 IU; vitamin E (all-rac-tocopherol acetate), 15 mg; vitamin K (bisulfate menadione complex), 1.2 mg; riboflavin, 4.4 mg; pantothenic acid (d-calcium pantothenate), 6.6 mg; niacin 21 mg; choline (choline chloride), 358 mg; vitamin B12 (cyanocobalamin), 0.006 mg; manganese (MnSO_4_·H_2_O), 83 mg; zinc (ZnO), 61 mg; iron (FeSO_4_·H_2_O), 32 mg; copper (CuSO_4_·5H_2_O), 3.9 mg; iodine (KI), 1.1 mg; selenium (Na_2_SeO_3_), 0.256 mg.

## Data Availability

The data are available upon reasonable request from the corresponding authors.
